# Comment: Is off-label medication use in the ICU a problem?

**DOI:** 10.1186/s13054-023-04546-y

**Published:** 2023-07-15

**Authors:** Menino O. Cotta, Jason A. Roberts, Michael C. Reade

**Affiliations:** 1 University of Queensland Centre for Clinical Research, Faculty of Medicine, University of Queensland, Herston, Brisbane, QLD Australia; 2 Pharmacy Department, Royal Brisbane and Women’s Hospital, Herston, Brisbane, QLD Australia; 3 Department of Intensive Care Medicine, Royal Brisbane and Women’s Hospital, Butterfield Street, Herston, Brisbane, QLD 4029 Australia; 4 Division of Anaesthesiology Critical Care Emergency and Pain Medicine, Nimes University Hospital, University of Montpellier, Nîmes, France; 5 Herston Infectious Diseases Institute (HeIDI), Metro North Health, Brisbane, Australia; 6 Medical School, University of Queensland, Herston, Brisbane, QLD Australia; 7 Joint Health Command, Australian Defence Force, Canberra, Australian Capital Territory Australia

‘Off-label’ medication use involves prescribing for an indication, route, or patient group not included in the approved product information. Between 25 and 33% of medications prescribed in the intensive care unit (ICU) are off-label [1–3]. Nearly all patients (88.0% in one study [3]) receive at least one off-label medication during their ICU stay. In a study of 327 ICU patients, although the rate of adverse drug reactions associated with off-label use was no different to that with licensed therapies, the incidence of adverse drug reactions increased by 8% with each additional off-label medication [4]. Several authors have argued that individual patient informed consent should be obtained whenever off-label medications are prescribed, [5, 6] noting potential harm from untested use, unknown drug interactions, possible unwarranted expense, and lack of governance [6]. Implementing this policy in the ICU, even with surrogate consent, would be impractical given the number of medications involved.

Nevertheless, there is often a rationale for off-label medication use among critically ill patients. This can include:

- indications similar to those in the product information (e.g. pantoprazole for stress ulcer prophylaxis rather than the approved indication of ulcer treatment)
- well-described pharmacological activity but trial evidence insufficient for regulatory approval (e.g. erythromycin as a prokinetic agent rather than the approved indication as an antibiotic; fentanyl at rates higher than those registered [in Australia, 100 mcg/h]) to facilitate mechanical ventilation
- preferable pharmacokinetics or dynamics when administered by a certain route (e.g. inhaled amikacin for gram-negative ventilator-associated pneumonia)
- alternative therapy when licensed treatment options have been exhausted (e.g. clonidine for delirium and high sympathetic activity during opioid or alcohol withdrawal, rather than the approved indication for hypertension; methylene blue or hydroxocobalamin for refractory vasodilatory shock in sepsis).

However, off-label prescribing with little regulatory oversight can also be problematic. E.g. empiric use of hydroxychloroquine as a treatment for COVID-19 based on extrapolation from in vitro data precipitated shortages for indicated uses [7]. Thus, there are challenges in balancing safe, evidence-based use of medications with clinical need at an individual patient level. If seeking individual patient or surrogate consent is impossible or even inadequate for the purpose of optimising patient safety, what alternatives might be available to assure critical care clinicians and their patients that prescribing decisions are sound?

First, implementing an effective audit loop at the hospital level to evaluate prescribing frequency and clinical effects would provide assurance that clinician practice is harmonised with discipline norms. This may be especially relevant with medications for which the risk / benefit is not clearly known, when costs are especially high, and when off-label use is sufficiently common to make such an audit meaningful. The audit framework should be part of a wider hospital multi-disciplinary drug evaluation process incorporating the pharmacy and hospital drug and therapeutics body [8]. Such routine prospective audits are less frequently reported in the literature than projects undertaken at a single timepoint, perhaps due to their cost, but the success of this approach has nonetheless been demonstrated. For example, concerned at possible inappropriate use of factor VIIa, a US hospital implemented an evidence-based guideline for off-label use. Routine audit demonstrated decreased mean dosages, overall utilisation, and cost [9]. Implementing effective oversight does not necessarily equate to requiring approval or informed consent for individual prescriptions, which might not be practical or appropriate for many critical care medications and/or indications. Rather, conducting these activities within the hospital governance structure allows for a cyclical evaluation, particularly applicable when off-label uses change periodically over time. An example is the treatment of ICU delirium, where many pharmacological interventions have been proposed and the landscape of off-label treatment is ever changing [10].

Second, national or international observational studies can inform understanding of what is considered acceptable practice by most critical care clinicians. An example is a study of dexmedetomidine dosage in Australian ICUs, which found clinicians frequently choosing maximum doses (up to 4.0 mcg/kg/h) exceeding the upper limit approved in registered product information (in Australia, 1.0 mcg/kg/h) and that commonly used in clinical trials (1.5 mcg/kg/h) [11]. The major barrier to reliance on this ad-hoc approach is the time taken to produce results with sufficient external validity: this dexmedetomidine study was highly topical, but still required 19 months to gather data from six of the 14 hospitals originally intended. Such observational studies may nonetheless form the basis for interventional trials and are often the first step in a research program seeking to optimise the effectiveness of off-label medication use.

A third important activity to consider, and one likely to be informed by observational studies, is the development of clinical guidelines that explicitly define acceptable off-label medication use. Through review of relevant literature, input from experts and endorsement by professional societies, published authoritative guidelines can provide details required by clinicians when prescribing medications for off-label use. A 2017 study determining the presence of guideline support for off-label medication use revealed > 50% off-label indications used in ICUs were specified in clinical guidelines [12], most with a high level of evidence. Although findings supported the view that clinicians should consider using guidelines to inform off-label medication use in the ICU, a considerable proportion of off-label use (44% in that study) occurs without any guideline support. Guidelines based on accumulated experience will never encompass all clinician-perceived requirements to prescribe off-label. Guidelines should only ever reflect accumulated clinical knowledge, which will take time to accrue. We do not suggest that an entirely new drug should be recommended by guidelines for widespread use in a manner other than that supported by registered trial evidence.

Lack of financial or regulatory incentives for pharmaceutical companies to expand licensed uses of their products has been noted as a reason that off-label use without robust evidence continues to occur [13, 14]. Ongoing engagement between clinicians and pharmaceutical companies may help breach these gaps and channel research into off-label medication use where it is most needed. To facilitate this engagement, a fourth strategy might be to lift the prohibition on pharmaceutical companies facilitating discussion of off-label uses of medications, as long as they explicitly disclose when this occurs. If discussion facilitated recognition of broad acceptance of safety and effectiveness within the clinical community, regulatory authorities might be persuaded to add indications, doses or routes to approved product information without requiring additional expensive trials that are unlikely to be performed.

In summary, there is scope to enhance monitoring and provide evidence for off-label medication use in the ICU. Sustainable strategies should provide feedback that continually informs clinical practice. These strategies should not cause unnecessary delays in medication administration. Specifically, calls to require individual patient or surrogate informed consent in critical care for medications that form part of accepted practice should be dismissed. Clinical research engaging multiple stakeholders including pharmaceutical companies may help target off-label medication uses for which evidence is currently most needed. Endorsed, evidence-based clinical guidelines reflecting accepted practice would overcome many of the hypothesised problems associated with off-label medication use, becoming an invaluable resource for clinicians and patients.

## Data Availability

Not applicable.
